# Development of a Fluorescent Based Immunosensor for the Serodiagnosis of Canine Leishmaniasis Combining Immunomagnetic Separation and Flow Cytometry

**DOI:** 10.1371/journal.pntd.0002371

**Published:** 2013-08-22

**Authors:** Susana Sousa, Luís Cardoso, Steven G. Reed, Alexandre B. Reis, Olindo A. Martins-Filho, Ricardo Silvestre, Anabela Cordeiro da Silva

**Affiliations:** 1 Parasite Disease Group, IBMC - Instituto de Biologia Molecular e Celular, Universidade do Porto, Porto, Portugal; 2 Departamento de Ciências Veterinárias, Universidade de Trás-os-Montes e Alto Douro, Vila Real, Portugal; 3 Infectious Disease Research Institute, Seattle, Washington, United States of America; 4 Laboratório de Imunopatologia, Núcleo de Pesquisas em Ciências Biológicas (NUPEB), Universidade Federal de Ouro Preto (UFOP), Campus Universitário Morro do Cruzeiro, Ouro Preto, Minas Gerais, Brazil; 5 Departamento de Análises Clínicas, Escola de Farmácia, Universidade Federal de Ouro Preto, Ouro Preto, Minas Gerais, Brazil; 6 Centro de Pesquisas René Rachou, FIOCRUZ, Belo Horizonte, Minas Gerais, Brazil; 7 Departamento de Ciências, Instituto Superior de Ciências da Saúde - Norte, CESPU, CRL, Gandra, Portugal; 8 Departamento de Ciências Biológicas, Faculdade de Farmácia, Universidade do Porto, Porto, Portugal; Institut Pasteur de Tunis, Tunisia

## Abstract

**Background:**

An accurate diagnosis is essential for the control of infectious diseases. In the search for effective and efficient tests, biosensors have increasingly been exploited for the development of new and highly sensitive diagnostic methods. Here, we describe a new fluorescent based immunosensor comprising magnetic polymer microspheres coated with recombinant antigens to improve the detection of specific antibodies generated during an infectious disease. As a challenging model, we used canine leishmaniasis due to the unsatisfactory sensitivity associated with the detection of infection in asymptomatic animals where the levels of pathogen-specific antibodies are scarce.

**Methodology:**

Ni-NTA magnetic microspheres with 1,7 µm and 8,07 µm were coated with the *Leishmania* recombinant proteins *Li*cTXNPx and rK39, respectively. A mixture of equal proportions of both recombinant protein-coated microspheres was used to recognize and specifically bind anti-rK39 and anti-*Li*cTNXPx antibodies present in serum samples of infected dogs. The microspheres were recovered by magnetic separation and the percentage of fluorescent positive microspheres was quantified by flow cytometry.

**Principal Findings:**

A clinical evaluation carried out with 129 dog serum samples using the antigen combination demonstrated a sensitivity of 98,8% with a specificity of 94,4%. rK39 antigen alone demonstrated a higher sensitivity for symptomatic dogs (96,9%), while *Li*cTXNPx antigen showed a higher sensitivity for asymptomatic (94,4%).

**Conclusions:**

Overall, our results demonstrated the potential of a magnetic microsphere associated flow cytometry methodology as a viable tool for highly sensitive laboratorial serodiagnosis of both clinical and subclinical forms of canine leishmaniasis.

## Author Summary

Dogs are the most important domestic reservoirs of the parasite *Leishmania infantum*. Some infected animals develop a subclinical infection, without the classical symptoms characteristics of this disease. One of the major challenges in the serodiagnosis of canine leishmaniasis is the detection of actively infected animals that are already able to transmit the parasite to the vector, despite the fact they did not yet show external signs of the disease. In the present work, we have developed a new tool for the laboratorial diagnosis of canine leishmaniasis that clearly improves the performance of canine leishmaniasis serodiagnosis. An immunofluorescence assay was developed combining *Leishmania* recombinant protein-coated magnetic microspheres and flow cytometry. The antigen-coated microspheres were used to separate anti-*Leishmania* specific antibodies present in the serum of infected dogs. Flow cytometry allowed the specific quantification of the antibodies. The clinical evaluation of a panel of serum samples from natural infected dogs clearly demonstrates that this method detects with high specificity and sensitivity both clinical and subclinical forms of the disease.

## Introduction

Efficient diagnostic tests capable of providing early and accurate diagnosis are essential in determining the choice of treatment and in the epidemiological surveillance of infectious diseases. Classically, the microscopic observation or isolation of the infectious agent was considered as the gold standard for laboratory confirmation of an infection. During the last decades, the development of molecular biology techniques capable of detecting and quantifying pathogen-specific DNA or RNA have emerged [1]. Despite their high sensitivity, these techniques often require specific and expensive equipment and highly trained personnel. On the other hand, serological approaches to detect specific antibodies against an infectious agent constitute a valuable alternative for early, rapid, and user-friendly diagnostic tests for both human and veterinary infections. The use of defined and well-characterized recombinant antigens has improved the performance of serodiagnosis in several infectious diseases by increasing overall sensitivity and specificity [2], [3], [4]. The last few years have positioned flow cytometry analysis as an emerging technology for the diagnosis of infectious diseases [5]. This technique possesses several advantages for immunoassays such as high throughput capacity, possibility of analyte quantification, reduced sample volume, high reproducibility and sensitivity, a wide dynamic range, and, the most exciting of all, the potential for multiplexing [5]. More recently, micro and nanotechnology have been applied in the development of biosensors that emerge as promising diagnostic methods [6]. Microsphere-based immunoassays with covalent binding between an antigen or antibody to magnetic microspheres have been considered promising alternatives for serological analysis [7].

Leishmaniasis is a zoonotic disease caused by protozoa of the genus *Leishmania*. Dogs are considered the main reservoir hosts for the zoonotic cycle of this parasite. Canine leishmaniasis (CanL) is a systemic chronic disease, ranging from asymptomatic subclinical to symptomatic infection. Nevertheless, actively infected animals, despite they did not show yet external signs of the disease [8] are already able to transmit the parasite to the vector, the phlebotomine sand flies [9]. As a consequence, CanL represents an important veterinary and public health problem since it contributes to the maintenance of the *Leishmania* life cycle and transmission to humans. As a result, the development of specific and efficient diagnostic methods capable of detecting both symptomatic and asymptomatic infected animals is essential for the control of this zoonosis, with special attention being paid to the unsatisfactory sensitivity associated with the detection of subclinical infections [10].

The present work describes a new method for the serodiagnosis of canine leishmaniasis. This method combines antigen-coated magnetic microspheres, immunomagnetic separation and flow cytometry for the detection of specific antibodies to *Leishmania*. An immunofluorescent assay was developed using a mixture of two magnetic microspheres with distinguishable size coated with the *Leishmania* recombinant proteins rK39 and *Li*cTXNPx, which were recently proved to be a useful tool for the detection of both clinical and subclinical forms of canine *Leishmania* infection [11]. After magnetic separation, positive fluorescent microspheres were quantified by flow cytometry. A clinical evaluation of the method was done using a panel of serum samples from natural infected dogs.

## Methods

### Ethics statement

This study observed Portuguese legislation for the protection of animals (Law no. 92/1995, from September 12th). According to the European Directive of 24 November 1986, article 2 d, non experimental, agricultural or clinical veterinary were excluded. The Animal Ethics Committee of the Associate Laboratory IBMC-INEB approved the animal protocol used. Serum samples were collected during vaccination campaigns and informed consent was obtained from all dog owners before sample collection.

### Animal samples

129 serum samples from domestic dogs were used in this work. Dogs were clinically classified as symptomatic, asymptomatic and healthy dogs. Sera from *Leishmania*-negative dogs presenting non-related pathologies were used as controls for cross-reactivity. Peripheral blood was collected from the cephalic vein and stored at −20°C. Serology for antibodies to *Leishmania* was performed by Direct Agglutination Test (DAT) according to the protocol described by Schallig et al [12]. For parasitological studies, bone marrow or lymph node aspirates were collected for microscopic examination. For PCR, DNA was extracted from blood.

Based on the clinical, serological and parasitological examination, animals were divides into four groups:

1. 32 serum samples from symptomatic dogs, as defined by the presence of at least two clinical signs compatible with CanL. Animals from this group were seropositive for anti-*Leishmania* antibodies (DAT titre>1∶400) and parasitologically positive.
2. 31 serum samples from asymptomatic dogs, living in endemic areas for CanL, but with no history of CanL. These animals were seropositive for anti-*Leishmania* antibodies (DAT titre>1∶400)
3. 18 serum samples from asymptomatic dogs, living in endemic areas for CanL, seronegative for anti*-Leishmania* antibodies (DAT titre<1∶400), but positive by PCR.
4. 36 serum samples from clinically healthy dogs from non-endemic areas, seronegative for *Leishmania* (DAT titre<1∶400) and parasitologically negative.
5. 12 serum samples from dogs from endemic areas for CanL, seronegative for *Leishmania* (DAT titre<1∶400) and parasitologically negative but infected with other agents (*Ancylostoma caninum*, *Babesia canis* and *Leptospira* spp. mixed infection, *Leptospira* spp., *Dipylidium caninum*, *Taenia* spp., *Toxocara canis* and *Sarcoptes scabiei* mixed infection, *Demodex canis*, *Hepatozoon canis*, *Trichuris vulpis*, tick infestation, autoimmune disorder, lymphoma, pneumonia, pyodermitis and tumor).

### Antigens

Two recombinant proteins, *L. infantum* cytosolic tryparedoxin peroxidase (*Lic*TXNPx) and rK39 [13] were used in the present study. *Li*cTXNPx was prepared as described by Cordeiro-da-Silva et al [14]. Both proteins contain a six-histidine residue at its N-terminal.

### Preparation of antigen-coated magnetic microspheres

Superparamagnetic silica microspheres coated with Ni-NTA as the functional group with two different sizes (8.07 µm and 1.7 µm) were specifically synthesized for this study by Kisker-biotech (Germany). Both microspheres (5×10^6^) were coated with 5 µg of rk39 or *Li*cTXNPx through binding of the recombinant protein histidine tail to the Ni-NTA groups present on the surface of the microspheres.

### Preparation of samples for flow cytometry

Recombinant protein-coated microspheres were blocked for 1 h at 37°C with PBS containing one of the following blocking agents: 5% non-fat milk, 3% gelatin, 10% heat inactivated fetal bovine serum (FBS) or 3% bovine serum albumin (BSA). The microspheres were separated using a neodymium magnet separation rack and washed twice in PBS. After blocking, the coated magnetic microspheres were mixed in an equal proportion (50%∶50%) in a final volume of 100 µl. The mixture was incubated with 100 µl of dog serum sample dilutions ranging from 1∶100 to 1∶6400 in PBS and incubated for 30 minutes at room temperature with gentle mixing. After a washing step, the mixture was incubated with 100 µl FITC-conjugated sheep anti-dog IgG diluted at 1∶100 for 30 minutes at room temperature in the dark with gentle mixing. The microspheres were then recovered by magnetic separation and washed three times in PBS and resuspended in 500 µl of PBS.

### Flow cytometry

The microspheres were analyzed by flow cytometry in a FACSCalibur and analyzed with FlowJo software. Internal controls of the reaction were included in all experiments to monitor unspecific binding, in which the microspheres were incubated in the absence of dog serum, but in the presence of FITC conjugated goat anti-dog IgG. Also, in all batches of the experiments, positive and negative controls were included. Recombinant protein-coated microspheres were identified on the basis of forward/side scatter values. Gated cells (excluding duplets) were evaluated by FL-1 area versus forward scatter (FSC) pattern and a total of 10,000 events were acquired.

### Statistical analysis

Statistical analysis was performed using GraphPad Prism 5 software. Differences in immunoglobulin levels between groups were analyzed by means of the Mann–Whitney's test. A P-value<0.05 was considered as statistically significant.

## Results

### Establishment of the experimental protocol

**Figure 1:**
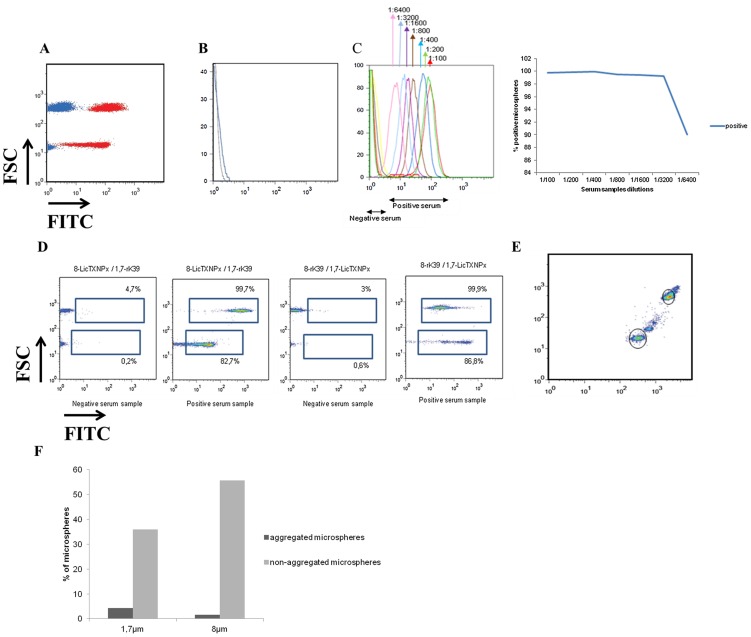
Optimization of the experimental conditions. (A) Unspecific binding of serum antibodies to uncoated magnetic microspheres. Positive serum sample is represented in red, while negative serum sample in blue. (B) BFS as blocking agents (blue for positive serum and black for negative). (C) Positive and negative serum samples diluted from 1∶100 to 1∶6400. (D) PMSI-8.07Ni-NTA coated with rK39 and PMSI-1.7Ni-NTA coated with *Li*cTXNPx in the presence of a positive serum or a negative serum and PMSI-8.07Ni-NTA coated with *Li*cTXNPx and PMSI-1.7Ni-NTA coated with rK39 in the presence of a positive serum or a negative serum. (E) PMSI-8.07Ni-NTA coated with rK39 and PMSI-1.7Ni-NTA coated with *Li*cTXNPx formed populations of different sizes as consequence of microspheres aggregation. For further analysis, only the non-aggregated populations (black line) were used. (F) Percentage of aggregated microspheres is higher in PMSI-1.7Ni-NTA than in PMSI-8.07Ni-NTA microspheres.

Our initial hypothesis for the development of a fluorescent based immunosensor started for binding sensitive and specific defined antigens, already validated, to the magnetic microspheres. Nevertheless, unspecific adsorption of sera antibodies to the magnetic microspheres was observed when uncoated microspheres were incubated with a positive serum sample (Figure 1A). To eliminate the unspecific binding of serum antibodies to the uncoated microspheres, several proteins were tested as reported in Methods. These proteins have been described as blocking agents of immunoassays such as ELISA and Western-blot with the optimal blocking agent for any particular assay to be determined by empirical testing. Uncoated magnetic microspheres coated with 3% gelatin still recognized positive serum samples, but the majority of the microspheres were lost during the assay (data not shown). When using 5% non-fat milk or 3% BSA, 99% of the microspheres were found to have positive signal in the presence of a positive serum sample (data not shown). FBS-coated microspheres, in the presence of positive or negative serum samples, showed similar results with very low percentage of positive microspheres (Figure 1B). The next step was to determine the optimal serum dilution. For that, serial dilutions ranging from 1∶100 to 1∶6400 were tested in recombinant protein-coated microspheres blocked with 10% FBS (Figure 1C). A good distinction between positive and negative serum samples was achieved up to 1∶3200. Microspheres with two different sizes, each one coated with rk39 or *Li*cTXNPx were used for the development of this immunofluorescent assay. This will allow a clear separation of these microspheres by flow cytometry and consequently specifically quantify anti-rK39 and anti-*Li*cTXNPx antibodies. A better separation between positive and negative serum samples was obtained when larger magnetic microspheres (PMSI-8.07Ni-NTA) were coated with the recombinant protein rK39 and the smaller ones (PMSI-1.7Ni-NTA) coated with the recombinant protein *Li*cTXNPx as shown in Figure 1D. It was observed that the magnetic microspheres when coated with these *Leishmania-*specific proteins formed two or more populations (Figure 1E), which was found to be more evident for the low size magnetic microspheres (Figure 1F). We believe that the larger populations are the result of microspheres aggregation, which lead us to exclude these smaller populations of the analysis.

### Immunofluorescent assay combines rK39 and *Li*cTXNPx-coated magnetic microspheres

**Figure 2:**
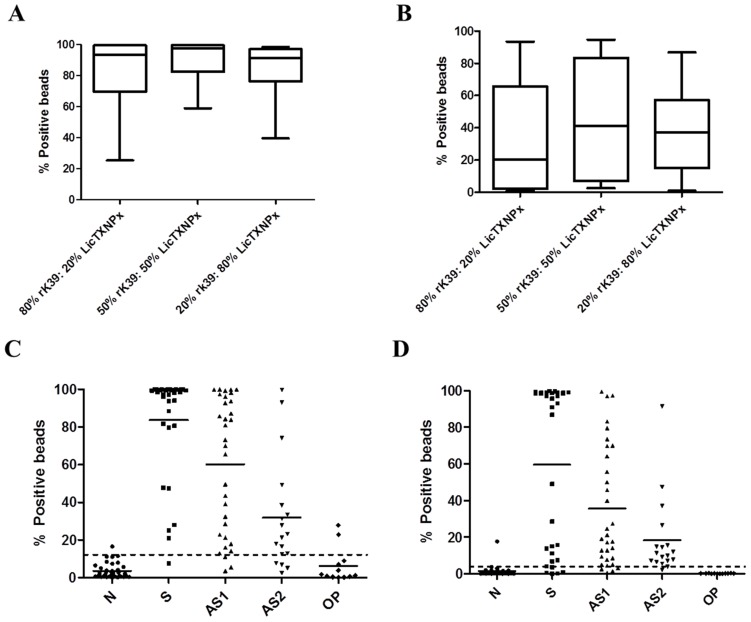
Magnetic microspheres flow cytometry characterization and evaluation. Reactivities of representative symptomatic (A) and asymptomatic (B) sera tested to different defined combinations of *Li*cTXNPx and rk39 antigens. Results are expressed as the percentage of positive microspheres. The levels of IgG antibodies anti-rK39 (C) and anti-*Li*cTXNPx (D) were measured in sera of symptomatic (S), asymptomatic (AS1), asymptomatic PCR+ (AS2), *Leishmania*-negative but presenting other clinical conditions (OP) and *Leishmania* negative healthy dogs from non-endemic areas (N). Results are expressed as the percentage of positive microspheres.

Based on these previous results, we have applied the same principle of combining these two proteins to develop this new method. In order to determine the optimal combination of rK39 and *Li*cTXNPx, we determined the reactivity of randomly chosen 20 symptomatic and asymptomatic serum samples against different combinations of these two proteins. The LAM-ELISA described by Santarém et al., [11] was described as the conjunction of 80% rK39 and 20% *Li*cTXNPx. Thus, we have selected this proportion along with a 50% rK39: 50% *Li*cTXNPx and 20% rK39: 80% *Li*cTXNPx mixtures. As shown in Figure 2A and B, 50% rK39: 50% *Li*cTXNPx achieved the higher percentage of fluorescent positive microspheres for both symptomatic and asymptomatic dogs. Negative dogs showed no significant reactivity with the three different proportions (data not shown). Based on the results from Santarém et al [11] it was to be expected that the combination 80% rK39: 20% LicTXNPx would give the best results. During the immunoassay method, the magnetic microspheres are separated at several points of the protocol using a magnet. During these separating steps, a percentage of magnetic microspheres are lost, with higher incidence for the smaller microspheres. Since these smaller microspheres are coated with *Li*cTXNPx, a higher amount of these microspheres must be used to compensate the *Li*cTXNPx-coated microspheres lost during the process.

### Determination of cut-off values

On the basis of the histogram representing the binding of non-infected animals, an area was chosen in order to contain a maximum of 1% of fluorescent positive microspheres for each antigen in any negative sample. This area will be used to measure the percentage of fluorescent positive microspheres in all data. The cut-off, defined by the ROC curve for rK39 antigen, corresponded to 12.2% of fluorescent positive microspheres. The area under the curve was 0.9857, 95% confidence interval: 0.9693–1.002. The cut-off, defined by the ROC curve for *Li*cTXNPx antigen, was 3.9% of fluorescent positive microspheres. The area under the curve was 0.9366, 95% confidence interval: 0.8885–0.9847 [15].

### Combination of rK39 and *Li*cTXNPx-coated microspheres increases sensitivity of the immunofluorescent assay

The magnetic immunoassay method was applied to the diagnostic of CanL. Using the established optimal conditions, a panel of 129 serum samples was studied. Immunofluorescent assay was considered positive whenever at least one antigen was positive. With the defined cut-offs, a sensitivity of 91.4% was achieved for rK39 and a sensitivity of 88.9% for *Li*cTXNPx with a specificity of 97.2% for both antigens (Figure 2C and 2D). Comparing the results obtained with symptomatic and asymptomatic dogs, rK39 antigen demonstrated a higher sensitivity for symptomatic dogs (96.8%) than for asymptomatic animals (93.5% for group 2 and 77.8% for group 3), while *Li*cTXNPx antigen showed a higher sensitivity for asymptomatic (90.3% for group 2 and 94.4% for group 3) than for symptomatic dogs (84.3%). Together these antigens increase the sensitivity of the immunofluorescent assay to 98.8% (Table 1). The use of *Leishmania* recombinant antigens is less prone to cross-reactivity, displaying lower false-positive reactions [16]. Cross-reactivity of magnetic microspheres flow cytometry was evaluated using 12 serum samples from dogs seronegative for *Leishmania*, but with other clinical conditions (group 5). Only two out of twelve serum samples cross-reacted with rK39-coated beads (Figure 2C). A low level of cross-reactivity was also reported for LAM-ELISA [11].

**Table 1:** Sensitivity and specificity of the immunofluorescent assay in the diagnosis of *Leishmania* infected dogs.

Antigen	Sensitivity^a^				Specificity^b^
	Symptomatic dogs	DAT-positive asymptomatic dogs	DAT-negative PCR+ asymptomatic dogs	Total^c^	
rK39	31/32 (96,8)	29/31 (93,5)	14/18 (77,8)	74/81 (91,4)	35/36 (97,2)
*Lic*TXNPx	27/32 (84,3)	28/31 (90,3)	17/18 (94,4)	72/81 (88,9)	35/36 (97,2)
rK39+*Lic*TXNPx	32/32 (100)	31/31 (100)	17/18 (94,4)	80/81 (98,8)	34/36 (94,4)

a [true positives/(true positives+false negatives)] (percentage).

b [true negatives/(true negatives+false positives)] (percentage).

c Symptomatic and asymptomatic.

## Discussion

We have recently proposed a defined *Leishmania* antigen mixture, composed of the *Li*cTXNPx and rK39 antigens, as an improvement to current ELISA-based serological techniques for the accurate detection of both clinical and subclinical forms of CanL [11]. The combined use of these two antigens achieved the highest score in both symptomatic and asymptomatic dogs among all antigens used. The aim of the present work was to develop a magnetic immunosensor incorporating these antigens that when associated with flow cytometry could be used as a valid approach for the serodiagnosis of CanL. Therefore, magnetic polymer microspheres were coated with the two recombinant antigens of *Leishmania*. Antibodies present in positive serum samples will recognize and interact with these antigen-modified microspheres. Finally, the complex antibody-antigen-magnetic microspheres will be captured by a neodymium magnet and positive microspheres will be quantified by flow cytometry. The principle of using magnetic microspheres for the development of diagnosis methods has been explored due to the ability of these scaffolds to easily adsorb biological materials such as proteins, antibodies or DNA [17], [18]. We and others have already proposed the use of flow cytometry-based methods for the diagnosis of CanL using both promastigote as well as amastigote forms [19], [20]. The development of fluorescent based immunosensors by coupling highly sensitive flow cytometry to protein-coated magnetic polymer microspheres capable of specifically retain the target antibodies was anticipated to increase overall performance without the problematic of using live or fixed parasites.

In the absence of a gold standard to integrate the results obtained with magnetic microspheres coupled with flow cytometry, these were analyzed using ROC curves to determine the theoretical cut-off values. The antigens used in this technique enable good predictive values for the study cohort with a AUC of 0,9366 and 0,9857 for *Lic*TXNPx and rK39 respectively. LAM-ELISA described by Santarém et al [11] had a AUC of 0,984. This allowed the comparison of the performances between the two methods with acceptable confidence. LAM-ELISA described by Santarém et al [11] showed a specificity of 96,3% and a sensitivity of 90,7%. In the present study, magnetic microspheres flow cytometry reached similar specificity (94,4%) but higher sensitivity (98,8%). Similarly to ELISA, this method showed a better performance when combining both antigens. Magnetic microspheres flow cytometry using rK39 as antigen showed a sensitivity of 91,4% and using *Li*cTXNPx as antigen showed a performance of 88,9%. These results not only confirmed *Li*cTXNPx as a good marker for the detection of asymptomatic infected dogs but, more importantly, allowed an increase in test performance in the detection of both asymptomatic and symptomatic dogs using the previous described antigens. With this immunofluorescent assay, we achieved to detect 48 out of 49 asymptomatic animals with high specificity (94,4%). Magnetic microspheres flow cytometry showed a sensitivity of 94,4% for the detection of infected animals seronegative by DAT (group 3). This method proved to be as good as other conventional serological methods to evaluate seropositive animals. More importantly, the developed method proved to be highly sensitive in detecting infected animals that are considered seronegative by conventional serological methods. Although being a serological method, magnetic microspheres flow cytometry cannot be considered a rapid and user friendly method. However, since this method allows the detection of infected animals that are seronegative by other conventional methods, we hypothesized that it can be a valuable alternative to conventional serological methods for the detection of *Leishmania* infected animals.

In conclusion this study reports the development of a new tool for the laboratorial diagnosis of CanL. The method here described explores the potential of flow cytometry as a diagnostic method associated with antigen-modified magnetic microspheres. So far, all the approaches using flow cytometry used promastigotes or amastigotes as targets to detect *Leishmania* specific antibodies [19], [20], [21]. Here, we propose the use of recombinant antigens as a better target to detect specific anti-*Leishmania* antibodies. Two *Leishmania* specific antigens, previously described as highly sensitive for the detection of symptomatic and asymptomatic infected dogs were selected to coat magnetic microspheres with two distinct sizes. These antigen-coated microspheres mixed in the proportion 50% rK39: 50% *Li*cTXNPx were used to separate anti-*Leishmania* specific antibodies present in the serum of infected dogs. Finally, flow cytometry allowed the specific quantification of the antibodies against anti-rK39 and anti-*Li*cTXNPx.

The magnetic microspheres associated flow cytometry clearly improved the performance of CanL serodiagnosis, detecting with high specificity and sensitivity both clinical and subclinical forms of CanL.

## Supporting Information

Checklist S1 STARD checklist for magnetic microspheres flow cytometry applied to the serodiagnosis of CanL.
(DOC)

Flowchart S1 STARD flowchart for magnetic microspheres flow cytometry applied to the serodiagnosis of CanL.
(DOC)
