# Supplementary material for: Development of a Fluorescent Based Immunosensor for the Serodiagnosis of Canine Leishmaniasis Combining Immunomagnetic Separation and Flow Cytometry
Source: PLoS Negl Trop Dis. 2013 Aug 22;7(8):e2371. doi: 10.1371/journal.pntd.0002371 (PMC3749986; doi:10.1371/journal.pntd.0002371)
Supplement: Checklist S1 — STARD checklist for magnetic microspheres flow cytometry applied to the serodiagnosis of CanL. (DOC) [file pntd.0002371.s001.doc]

**Checklist S1**

| **Section and Topic** | **Item**  **#** |  | **On page #** |
| --- | --- | --- | --- |
| TITLE/ABSTRACT/  KEYWORDS | 1 | To determine the sensitivity and specificity of a new fluorescent based immunosensor combining immunomagnetic separation and flow cytometry for the diagnosis of canine leishmaniasis. | 1, 2 |
| INTRODUCTION | 2 | An accurate diagnosis is essential for the control of infectious diseases. In the search for effective and efficient tests, biosensors have increasingly been exploited for the development of new and highly sensitive diagnostic methods. Here, we describe a new fluorescent based immunosensor comprising magnetic polymer microspheres coated with recombinant antigens to improve the detection of specific antibodies for leishmaniasis. | 3-5 |
| METHODS |  |  |  |
| *Participants* | 3 | The study population: Serum samples obtained from domestic dogs of northeastern Portugal in the years 2008 and 2009. | 6 |
|  | 4 | Participant recruitment: Animals were recruited based on their clinical condition (presence or absence of clinical signs compatible with canine leishmaniasis), on serological (Direct Agglutination test) and parasitological results. | 6 |
|  | 5 | Participant sampling: the study population included groups of dogs defined by selection criteria in items 3 and 4. | 6 |
|  | 6 | Data collection: data collection was planned after the index test and reference standard were perfomed (retrospective study). | 6 |
| *Test methods* | 7 | Direct Agglutination Test was used as a standard method for serological diagnosis | 6 |
|  | 8 | Two recombinant proteins were used: *Lic*TXNPx and rK39. Superparamagnetic silica microspheres with Ni-NTA as the functional group of two different sizes were coated with the recombinant proteins. Recombinant protein-coated microspheres were incubated with serum samples of the dogs included in the study. Antibodies anti-rK39 and anti- *Lic*TXNPx present in the serum samples will recognize the proteins at the surface of the microspheres. Finally, the microspheres were analyzed by flow cytometry. | 7, 8 |
|  | 9 | Two cut-off values were defined, one for rK39 and one for *Lic*TXNPx | 10 |
|  | 10 | The number, training and expertise of the persons executing and reading the index tests and the reference standard. |  |
|  | 11 | Whether or not the readers of the index tests and reference standard were blind (masked) to the results of the other test and describe any other clinical information available to the readers. |  |
| *Statistical methods* | 12 | Differences in immunoglobulin levels between groups were analyzed by means of the Mann–Whitney’s test. | 8 |
|  | 13 | Methods for calculating test reproducibility, if done. |  |
| RESULTS |  |  |  |
| *Participants* | 14 | Beginning and end dates of recruitment: 1 April 2008 and 31 July 2009, respectively. |  |
|  | 15 | The spectrum of clinical signs in the symptomatic dogs was consistent with those of canine leishmaniosis or other diseases, depending on the group. | 6 |
|  | 16 | The number of participants satisfying the criteria for inclusion who did or did not undergo the index tests and/or the reference standard; All animals included in the study undergo both test |  |
| *Test results* | 17 | Samples were collected in the scope of a diagnostic process and sick animals were treated in due course. |  |
|  | 18 | Distribution of severity of disease (define criteria) in those with the target condition; other diagnoses in participants without the target condition. |  |
|  | 19 | The levels of IgG antibodies anti-rK39 (C) and anti-*Li*cTXNPx (D) were measured in sera of symptomatic (S), asymptomatic (AS1), asymptomatic PCR+ (AS2), *Leishmania*-negative but presenting other clinical conditions (OP) and *Leishmania* negative healthy dogs from non-endemic areas (N). Results are expressed as the percentage of positive microspheres.  C)    D) | 18 |
|  | 20 | Any adverse events from performing the index tests or the reference standard. |  |
| *Estimates* | 21 | Estimates of diagnostic accuracy and measures of statistical uncertainty (e.g. 95% confidence intervals). |  |
|  | 22 | How indeterminate results, missing data and outliers of the index tests were handled. |  |
|  | 23 | Sensitivity and specificity of the immunofluorescent assay for the diagnosis of canine leishmaniasis   | Antigen | Sensitivity^a^ | | | | Specificity^b^ | | --- | --- | --- | --- | --- | --- | | Symptomatic | Asymptomatic | Asymptomatic PCR+ | Total^c^ | | rK39 | 31/32 (96,8) | 29/31 (93,5) | 14/18 (77,8) | 74/81 (91,4) | 35/36 (97,2) | | *Lic*TXNPx | 27/32 (84,3) | 28/31 (90,3) | 17/18 (94,4) | 72/81 (88,9) | 35/36 (97,2) | | rK39 + *Lic*TXNPx | 32/32 (100) | 31/31 (100) | 17/18 (94,4) | 80/81 (98,8) | 34/36 (94,4) | | 18 |
|  | 24 | Estimates of test reproducibility, if done. | Not done |
| DISCUSSION | 25 | The principle of using magnetic microspheres for the development of diagnosis methods has been explored due to the ability of these scaffolds to easily adsorb biological materials such as proteins, antibodies or DNA… This method proved to be as good as other conventional serological methods to evaluate seropositive animals. More importantly, the developed method proved to be highly sensitive in detecting infected animals that are considered seronegative by conventional serological methods… The magnetic microspheres associated flow cytometry clearly improved the performance of CanL serodiagnosis, detecting with high specificity and sensitivity both clinical and subclinical forms of CanL. | 12-14 |
