# Supplementary material for: Development of a Fluorescent Based Immunosensor for the Serodiagnosis of Canine Leishmaniasis Combining Immunomagnetic Separation and Flow Cytometry
Source: PLoS Negl Trop Dis. 2013 Aug 22;7(8):e2371. doi: 10.1371/journal.pntd.0002371 (PMC3749986; doi:10.1371/journal.pntd.0002371)
Supplement: Flowchart S1 — STARD flowchart for magnetic microspheres flow cytometry applied to the serodiagnosis of CanL. (DOC) [file pntd.0002371.s002.doc]

**Flowchart S1**
